# Activation of PI3K/AKT and ERK MAPK signal pathways is required for the induction of lytic cycle replication of Kaposi's Sarcoma-associated herpesvirus by herpes simplex virus type 1

**DOI:** 10.1186/1471-2180-11-240

**Published:** 2011-10-27

**Authors:** Di Qin, Ninghan Feng, Weifei Fan, Xinting Ma, Qin Yan, Zhigang Lv, Yi Zeng, Jianzhong Zhu, Chun Lu

**Affiliations:** 1 State Key Laboratory of Reproductive Medicine; 2 Key Laboratory of Pathogen Biology of Jiangsu Province; 3 Department of Microbiology and Immunology, Nanjing Medical University, 140 Hanzhong Road, Nanjing 210029, PR China; 4 Department of Urology, the First Affiliated Hospital of Nanjing Medical University, 300 Guangzhou Road, Nanjing 210029, PR China; 5 Department of Blood Tumor, Jiangsu Province Official Hospital, 65 Jiangsu Road, Nanjing 210024, PR China; 6 Department of Clinical Laboratory, Jiangsu Province Official Hospital, 65 Jiangsu Road, Nanjing 210024, PR China; 7 Department of Microbiology and Immunology, Youjiang Medical College for Nationalities, 98 Chengxiang Road, Bose 533000, PR China; 8 Cancer Virology Program, University of Pittsburgh Cancer Institute, Pittsburgh, PA 15232, USA

## Abstract

**Background:**

Kaposi's sarcoma-associated herpesvirus (KSHV) is causally linked to several acquired immunodeficiency syndrome-related malignancies, including Kaposi's sarcoma (KS), primary effusion lymphoma (PEL) and a subset of multicentric Castleman's disease. Regulation of viral lytic replication is critical to the initiation and progression of KS. Recently, we reported that herpes simplex virus type 1 (HSV-1) was an important cofactor that activated lytic cycle replication of KSHV. Here, we further investigated the possible signal pathways involved in HSV-1-induced reactivation of KSHV.

**Results:**

By transfecting a series of dominant negative mutants and protein expressing constructs and using pharmacologic inhibitors, we found that either Janus kinase 1 (JAK1)/signal transducer and activator of transcription 3 (STAT3) or JAK1/STAT6 signaling failed to regulate HSV-1-induced KSHV replication. However, HSV-1 infection of BCBL-1 cells activated phosphatidylinositol 3-kinase (PI3K)/protein kinase B (PKB, also called AKT) pathway and inactivated phosphatase and tensin homologue deleted on chromosome ten (PTEN) and glycogen synthase kinase-3β (GSK-3β). PTEN/PI3K/AKT/GSK-3β pathway was found to be involved in HSV-1-induced KSHV reactivation. Additionally, extracellular signal-regulated protein kinase (ERK) mitogen-activated protein kinase (MAPK) pathway also partially contributed to HSV-1-induced KSHV replication.

**Conclusions:**

HSV-1 infection stimulated PI3K/AKT and ERK MAPK signaling pathways that in turn contributed to KSHV reactivation, which provided further insights into the molecular mechanism controlling KSHV lytic replication, particularly in the context of HSV-1 and KSHV co-infection.

## 1. Background

Kaposi's sarcoma (KS) is a multifocal angioproliferative disease that often occurs in human immunodeficiency virus (HIV)-infected patients [1]. Now the accepted etiological agent of KS is KS-associated herpesvirus (KSHV)/human herpesvirus 8 (HHV-8) [2]. KSHV is also associated with another lymphoproliferative disorders: primary effusion lymphoma (PEL, also termed body cavity-based lymphoma, or BCBL) and multicentric Castleman's disease (MCD) [3]. All herpesviruses, including KSHV, display two patterns of infection: latent and lytic phases [4]. During latency, only a restricted set of viral genes is expressed. Upon induction of lytic infection, viral replication and transcription programs become fully activated, and new virions are packaged and released from the cells. Regulation of viral infection cycle is critical to the initiation and progression of KS. However, KSHV infection appears to be necessary but not sufficient for the development of KS without the involvement of other cofactors to reactivate KSHV lytic replication.

Previously, we demonstrated that both interleukin-4 (IL-4)/signal transducer and activator of transcription 6 (STAT6) and IL-6/Janus kinase 2 (JAK2)/STAT3 signal pathways modulated HIV-1 transactivative transcription protein (Tat)-induced KSHV replication [5]. Recently, we have also shown that herpes simplex virus type 1 (HSV-1) was another important cofactor that reactivated the lytic cycle replication of KSHV, and the production of IL-10 and IL-4 from HSV-1-infected BCBL-1 cells partially contributed to KSHV replication [6]. These facts led us to hypothesize that HSV-1 might reactivate KSHV lytic cycle replication by modulating multiple signal pathways of BCBL-1 cells on the basis of changing cellular cytokines protein expression profile [6].

To verify this hypothesis, in this study, we focused on the major pathways activated by IL-10/IL-10 receptor (R) and IL-4/IL-4R to evaluate their functions in HSV-1-induced KSHV lytic cycle replication. By transfecting a series of dominant negative mutants and protein expressing constructs and using pharmacologic inhibitors, we found that either IL-10/JAK1/STAT3 or IL-4/JAK1/STAT6 signaling was not involved in HSV-1-induced KSHV replication. However, activation of both phosphatidylinositol 3-kinase (PI3K)/protein kinase B (PKB, also called AKT) and extracellular signal-regulated protein kinase (ERK) mitogen-activated protein kinase (MAPK) signal pathways contributed to HSV-1-induced KSHV replication. These novel findings are believed to be the first report on the mechanisms of KSHV activation by HSV-1 and shed light on the pathogenesis of KSHV-induced malignancies.

## 2. Methods

### 2.1. Cell culture and virus infection

BCBL-1 cells (KSHV-positive and EBV-negative PEL cell lines) were obtained through acquired immunodeficiency syndrome (AIDS) Research and Reference Reagent Program, National Institutes of Health. Vero cells (African green monkey kidney fibroblasts) were obtained from American Type Culture Collection (ATCC). BCBL-1 and Vero cells were maintained in RPMI-1640 and Dulbecco's modified Eagle's medium (DMEM) respectively, both of which contained 10% fetal bovine serum (FBS), 2 mmol/l L-glutamine, 100 U/ml penicillin, and 100 μg/ml streptomycin at 37°C in a humidified, 5% CO_2 _atmosphere. HSV-1 (McKrae strain) was propagated and viral titers were determined in Vero cells as described previously [6]. The supernatant from normal Vero cells culture was used as a control (Mock). Before infection or transfection, BCBL-1 cells were incubated in serum-free RPMI-1640 medium for a maximum inducibility of KSHV replication [7].

### 2.2. Antibodies and reagents

Anti-phospho-STAT3 (Tyr705) rabbit monoclonal antibody (mAb), anti-phospho-PI3K p85 (Tyr458)/p55 (Tyr199) rabbit polyclonal antibody (pAb), anti-phospho-AKT (Ser473) mouse mAb, anti-phospho-GSK-3β (Ser9, GSK: glycogen synthase kinase) rabbit pAb, anti-phospho-c-Raf (Ser338) rabbit pAb, anti-phospho-MEK1/2 (Ser217/221, MEK: MAPK-ERK kinase) rabbit pAb, anti-phospho-ERK1/2 (Thr202/Tyr204) rabbit mAb, anti-STAT3 rabbit pAb, anti-PI3K p85 rabbit pAb, anti-GSK-3β rabbit mAb, anti-c-Raf rabbit pAb, anti-MEK1/2 rabbit pAb, anti-Flag M2 mouse mAb, anti-hemagglutinin (HA) rabbit mAb and LY294002 (PI3K inhibitor) were purchased from Cell Signaling Technologies (Beverly, MA, USA). Anti-PTEN (PTEN: phosphatase and tensin homologue deleted on chromosome ten) mouse mAb, anti-β-actin mouse mAb, anti-α-Tubulin mouse mAb, anti-GAPDH mouse mAb and horseradish peroxidase (HRP)-conjugated goat anti-mouse/rabbit IgG were obtained from Santa Cruz Biotechnologies (Santa Cruz, CA, USA). Anti-AKT rabbit pAb were obtained from BioVision (Mountain view, CA, USA). Anti-ERK1/2 rabbit pAb were obtained from Shanghai Kangchen Biotechnologies (Shanghai, China). Piceatannol (JAK1 inhibitor) was purchased from BIOMOL Research Laboratories Inc. (Plymouth Meeting, PA, USA). Both anti-phospho-STAT6 (Tyr641) mouse mAb and Peptide II (ERK inhibitor) were obtained from Calbiochem (Darmstadt, Germany). Anti-STAT6 rabbit pAb was purchased from Bethyl Laboratories Inc. (Montgomery, TX, USA). Anti-KSHV ORF59 mAb and viral IL-6 (vIL-6) rabbit pAb were obtained from Advanced Biotechnologies Inc. (Columbia, MD, USA). Anti-KSHV Rta (replication and transcription activator) antibody was generated by immunization of rabbits with ORF50 peptide (amino acids 667-691) [8].

### 2.3. Western blot analysis

After infection, cells were harvested and lysed in RIPA buffer containing protease and phosphatase inhibitors. 60-80 μg of proteins were loaded onto sodium dodecyl sulphate-polyacrylamide gel electrophoresis (SDS-PAGE), transferred to polyvinylidene fluoride (PVDF) membrane. The membrane was incubated with diluted primary Abs for overnight at 4°C, and then incubated with HRP-conjugated species-specific second Abs for 1 h at 37°C. Proteins were visualized by enhanced chemiluminescence (ECL) reagents (Cell Signaling Technologies) according to the manufacture's instructions.

### 2.4. RNA isolation and real-time quantitative PCR (RT-qPCR)

Total RNA was isolated from cells by using Trizol reagent (Invitrogen, Carlsbad, CA). RT-qPCR was performed in a GeneAmp 7300 sequence detection machine (Applied Biosystems, Foster City, CA) as described previously [9]. The sequences of KSHV ORF26 primer and probe were listed as described previously [9].

### 2.5. Plasmids and transfection

The dominant negative STAT3 construct (pMSCV-STAT3 dominant negative-GFP, abbreviated pST3-DN) was kindly provided by D. Link (Washington University School of Medicine, MO, USA) [10]. The dominant negative STAT6 construct (pDsRed1-N1-STAT6 dominant negative-RFP, abbreviated pST6-DN), containing amino acids 1-661 of STAT6, was a kind gift of K. Zhang (UCLA School of Medicine, CA, USA) [11]. The dominant negative construct of PI3K (P85σiSH2-N, designated as PI3K-DN in this study), the dominant negative construct of AKT (SRα-AKT, designated as AKT-DN), and corresponding control vectors pSG5 and pSRα were generously provided by B-H Jiang (Nanjing Medical University, Nanjing, China) [12]. The dominant negative MEK1/2 construct (MEK-DN) was presented as a gift by G. Chen (Medical College of Wisconsin, WI, USA). The protein expressing plasmid of GSK-3β (GSK-3β-S9A, there was a tag of HA) was purchased from Addgene (http://www.addgene.org). The PTEN cDNA plasmid (there was a tag of Flag) was constructed in our lab. BCBL-1 cells were electroporated at 250 V and 960 μF using a Gene Pulser (Bio-Rad Laboratories, Hercules, CA) as described elsewhere [13].

### 2.6. Detection of the release of KSHV progeny virions

After BCBL-1 cells were infected with HSV-1 for 48 h, supernatant from cell cultures was harvested and filtered through a 0.45-μm-pore-size filter. The filtered supernatant was centrifugated for 30 min at a speed of 15 000 rpm at 4°C and the precipitation contained KSHV progeny virions. The virions were resuspended in PBS and viral DNA was extracted using the high pure viral nucleic acid kit (Roche, Germany) as per the manufacturer's instructions. Purified viral DNA was used for real-time DNA-PCR analysis. The KSHV ORF26 gene cloned in the pcDNA3.1 (abbreviated pcDNA, Invitrogen) was used to generate the standard curve.

### 2.7. Immunofluorescence assay (IFA)

IFA was performed as described elsewhere [14]. Briefly, after HSV-1 infection, BCBL-1 cells were washed and smeared on chamber slides. Slides were incubated with a 1:100 dilution of anti-KSHV ORF59 mouse mAb. Alexa Fluor 568 (Invitrogen)-conjugated goat anti-mouse antibody (1:200 dilution) was used as a secondary antibody for detection. The cells were counterstained with 4','-diamidino-2-phenylindole. Images were observed and recorded with a Zeiss Axiovert 200 M epifluorescence microscope (Carl Zeiss, Inc.). Photographs of at least 10 unique fields were taken of every slide, and the number of positive and negative cells was counted separately by three individuals, including one who was blinded to the results to calculate the percentage of positive cells.

## 3. Results

### 3.1. Inhibition of JAK1/STAT3 and JAK1/STAT6 signal pathways does not affect HSV-1-induced KSHV lytic cycle replication

**Figure 1:**
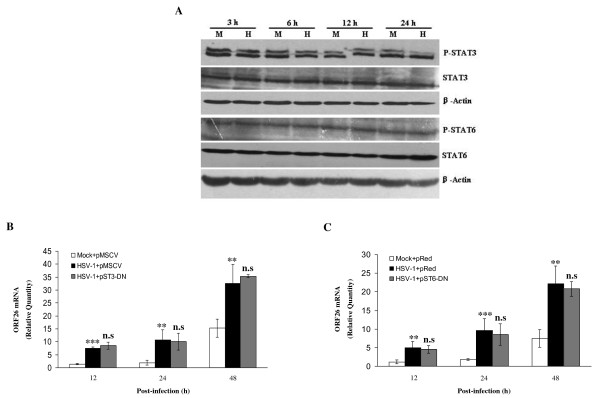
**Either JAK1/STAT3 or JAK1/STAT6 signal pathway does not mediate HSV-1-induced KSHV replication**. (A) Western blot analysis for phosphorylation of STAT3 and STAT6. BCBL-1 cells were infected with Mock (M) or HSV-1 (H) for 3, 6, 12, and 24 h. Cells were collected and cell lysates were subjected to SDS-PAGE, transferred to membrane, and then immunoblotted with the indicated antibodies. (B) RT-qPCR was used to detect relative quantities of ORF26 mRNA in STAT3-DN (pST3-DN) or control vector transfected and HSV-1 infected BCBL-1 cells as indicated. ** *p *< 0.01 and *** *p *< 0.001 for Student's t-test versus Mock + pMSCV group; n.s., not significant for Student's t-test versus HSV-1 + pMSCV group. (C) RT-qPCR was used to detect relative quantities of ORF26 mRNA in STAT6-DN (pST6-DN) or control vector transfected and HSV-1 infected BCBL-1 cells as indicated. ** *p *< 0.01 and *** *p *< 0.001 for Student's t-test versus Mock + pRed group; n.s., not significant for Student's t-test versus HSV-1 + pRed group.

We have previously demonstrated that the production of IL-10 and IL-4 from HSV-1-infected BCBL-1 cells partially contributed to HSV-1-induced KSHV replication [6]. Commonly, IL-10 exerts its function via JAK1, TYK2/STAT3 signal pathway, and IL-4 through JAK1, JAK3/STAT6 pathway [15-17]. To determine whether these signal pathways were altered in HSV-1-infected BCBL-1 cells, Western blot analysis was performed. As shown in Figure 1A, HSV-1 infection of BCBL-1 cells did not display any effect on phosphorylation of STAT3 or STAT6 at 3, 6, 12, and 24 h when compared to Mock-infected groups. Similar results were also observed when BCBL-1 cells were infected with HSV-1 or Mock at 15, 30, 45, and 60 min (data not shown). To confirm these results, BCBL-1 cells were transfected with STAT3-DN or STAT6-DN construct followed by HSV-1 infection. RT-qPCR demonstrated that transfection of either STAT3-DN or STAT6-DN did not affect KSHV ORF26 mRNA transcripts induced by HSV-1 in BCBL-1 cells (Figure 1B and 1C). To further extend above results, piceatannol, a JAK1 tyrosine kinase-specific inhibitor, was added to BCBL-1 cells culture before HSV-1 infection. The results from RT-qPCR indicated that inhibition of JAK1 did not influence KSHV replication by HSV-1 (data not shown). These data collectively suggest that either IL-10/JAK1/STAT3 or IL-4/JAK1/STAT6 signal pathway is not involved in HSV-1-induced KSHV replication.

### 3.2. Suppression of PI3K/AKT signal pathway inhibits HSV-1-induced KSHV replication

**Figure 2:**
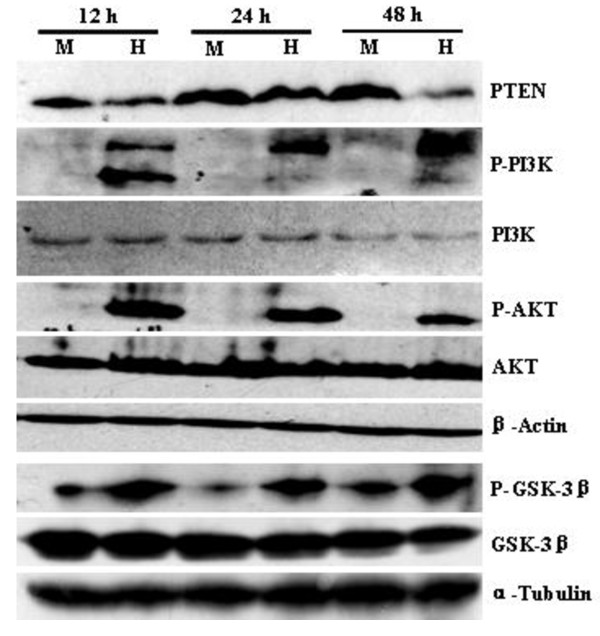
**Western blot analysis for phosphorylation of important molecules of PI3K/AKT pathway**. BCBL-1 cells were infected with Mock (M) or HSV-1 (H) for 12, 24, and 48 h. Cells were collected and cell lysates were subjected to SDS-PAGE, transferred to membrane, and then immunoblotted with the indicated antibodies.

Besides signal pathways from JAK1/STAT3 by IL-10 and JAK1/STAT6 by IL-4, both IL-10 and IL-4 can also induce activation of PI3K/AKT pathway [18-20]. To examine whether PI3K/AKT signaling was activated in HSV-1-infected BCBL-1 cells, Western blot analysis was carried out. It was demonstrated that phosphorylated PI3K and AKT were markedly increased at 12, 24, and 48 h in HSV-1-infected BCBL-1 cells when compared with Mock-infected BCBL-1 cells (Figure 2).

**Figure 3:**
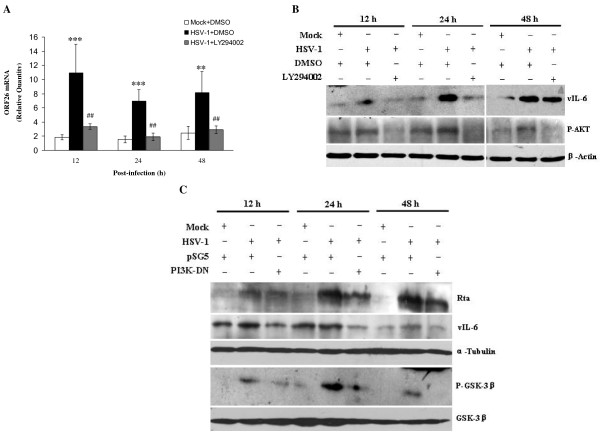
**Inhibition of PI3K suppresses HSV-1-induced reactivation of KSHV**. (A) RT-qPCR was used to detect relative quantities of ORF26 mRNA in LY294002 or DMSO control pretreated and HSV-1 infected BCBL-1 cells as indicated. ** *p *< 0.01 and *** *p *< 0.001 for Student's t-test versus Mock + DMSO group; ^## ^*p *< 0.01 for Student's t-test versus HSV-1 + DMSO group. (B) Western blot analysis was used to detect the expression of KSHV vIL-6 and phosphorylated AKT in LY294002 or DMSO pretreated and HSV-1 infected BCBL-1 cells as indicated. (C) Western blot analysis was used to detect the expression of KSHV Rta, vIL-6 and phosphorylated GSK-3β in PI3K-DN or control vector transfected and HSV-1 infected BCBL-1 cells as indicated.

**Figure 4:**
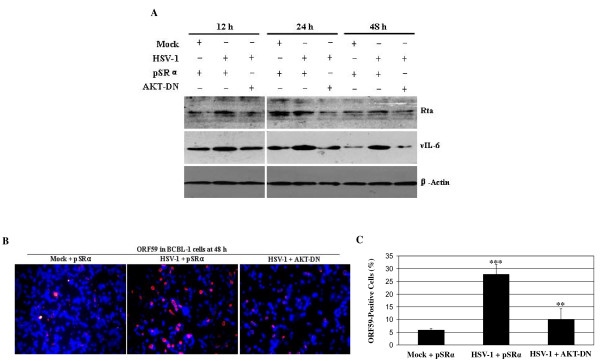
**Inhibition of AKT suppresses HSV-1-induced reactivation of KSHV**. (A) Western blot analysis was used to detect the expression of KSHV Rta and vIL-6 in AKT-DN or control vector transfected and HSV-1 infected BCBL-1 cells as indicated. (B) KSHV lytic proteins ORF59 expression in AKT-DN or control vector transfected and HSV-1 infected BCBL-1 cells was detected by IFA staining with ORF59 mAb. Original magnifications, × 10. (C) Quantification of results in B. *** *P *< 0.001 for Student's t-test versus Mock + pSRα group, whereas **P < 0.01 for Student's t-test versus HSV-1 + pSRα group.

To examine whether PI3K/AKT pathway was involved in KSHV lytic cycle replication by HSV-1, PI3K-specific inhibitor LY294002 was first used. RT-qPCR demonstrated that ORF26 mRNA in HSV-1-infected BCBL-1 cells pretreated with LY294002 was decreased 3.27-fold at 12 h, 3.64-fold at 24 h, and 2.81-fold at 48 h post infection of HSV-1, respectively, compared to HSV-1-infected BCBL-1 cells pretreated with DMSO (Figure 3A). To confirm this result, Western blot analysis was performed. We found that pretreatment of LY294002 inactivated the downstream kinase AKT and reduced the expression of KSHV vIL-6 proteins (Figure 3B). Next, PI3K-DN, the dominant negative form of PI3K, was transfected to BCBL-1 cells followed by HSV-1 infection. As shown in Figure 3C, control plasmid pSG5 alone did not affect KSHV activation by HSV-1, but transfection of PI3K-DN decreased HSV-1-induced KSHV Rta and vIL-6 expression. Finally, AKT-DN, the dominant negative form of AKT, was transfected to BCBL-1 cells followed by HSV-1 infection. Western blot analysis demonstrated that transfection of control plasmid pSRα alone did not influence KSHV replication, but transfection of AKT-DN down-regulated the proteins expression of KSHV Rta and vIL-6 (Figure 4A). The results from IFA also indicated that transfection of AKT-DN significantly decreased HSV-1-induced KSHV ORF59 proteins expression (Figure 4B and 4C). These data suggest that activation of PI3K/AKT pathway involves in HSV-1-induced KSHV replication.

### 3.3. Both overexpression of PTEN and activation of GSK-3β pathway also inhibit HSV-1-induced KSHV reactivation

**Figure 5:**
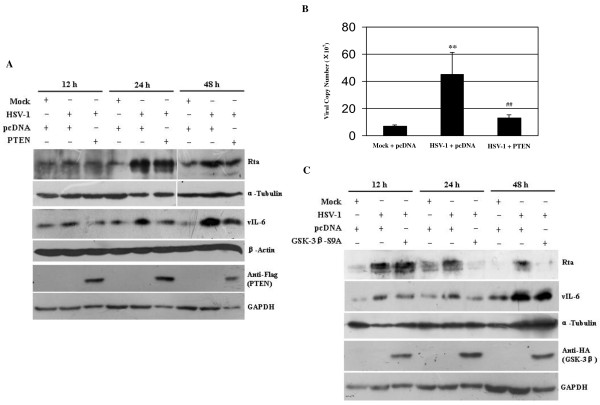
**Overexpression of PTEN and activation of GSK-3β inhibit HSV-1-induced KSHV reactivation**. (A) Western blot analysis was used to detect the expression of KSHV Rta, vIL-6 and the level of the transfected PTEN in PTEN or control vector transfected and HSV-1 infected BCBL-1 cells as indicated. (B) Real-time DNA-PCR was used to detect the copy number of KSHV progeny virions in the supernatant of PTEN or control vector transfected and HSV-1 infected BCBL-1 cells as indicated. ** *p *< 0.01 and ^## ^*p *< 0.01 for Student's t-test versus Mock + pcDNA and HSV-1 + pcDNA groups, respectively. (C) Western blot analysis was used to detect the expression of KSHV Rta, vIL-6 and the level of the transfected GSK-3β-S9A in GSK-3β-S9A or control vector transfected and HSV-1 infected BCBL-1 cells as indicated.

From Figure 2, we observed that expression of PTEN (negative regulator of PI3K/AKT pathway) was low in HSV-1-infected BCBL-1 cells, therefore, we asked whether overexpression of PTEN could influence HSV-1-induced KSHV replication. To address this issue, the PTEN cDNA construct was transfected to the cells. Western blot analysis demonstrated that overexpression of PTEN not only decreased phosphorylated AKT and GSK-3β (data not shown), but also reduced HSV-1-induced KSHV Rta and vIL-6 proteins expression (Figure 5A). To further determine whether overexpression of PTEN could reduce the release of KSHV progeny virions induced by HSV-1, experiments were designed to detect the copy number of KSHV progeny virions. The results of real-time DNA-PCR demonstrated that the copy number of KSHV virions in the supernatant from PTEN-transfected and HSV-1 infected BCBL-1 cells was significantly decreased when compared to those from pcDNA-transfected and HSV-1 infected BCBL-1 cells (Figure 5B).

Because HSV-1 infection of BCBL-1 cells increased phosphorylated GSK-3β (Figure 2) and transfection of PI3K-DN decreased HSV-1-induced phosphorylation of GSK-3β (Figure 3C), we reasoned that inactivated GSK-3β might promote HSV-1-induced KSHV replication. To test this hypothesis, the GSK-3β mutant plasmid GSK-3β-S9A, which exhibits constitutively active GSK-3β, was transfected to BCBL-1 cells. As expected, the expression of KSHV Rta and vIL-6 proteins in GSK-3β-S9A-transfected and HSV-1 infected BCBL-1 cells was markedly reduced compared to pcDNA-transfected and HSV-1 infected BCBL-1 cells (Figure 5C).

Taken together, these data suggest that PTEN/PI3K/AKT/GSK-3β pathway may play an important role in HSV-1-induced KSHV reactivation.

### 3.4. ERK MAPK pathway partially contributes to HSV-1-induced KSHV replication

**Figure 6:**
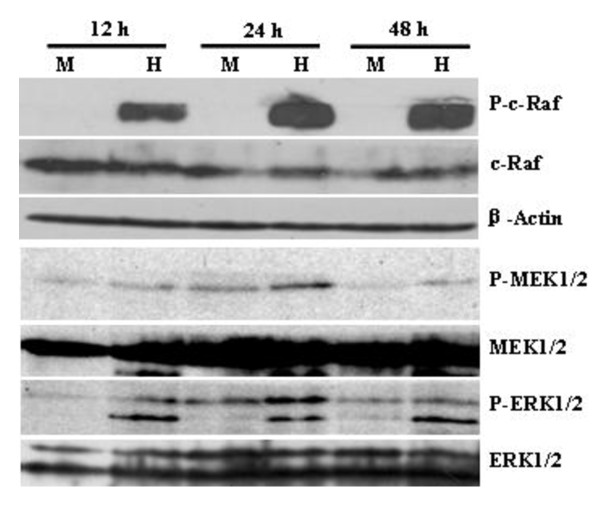
**Western blot analysis for phosphorylation of important molecules of ERK MAPK pathway**. BCBL-1 cells were infected with Mock (M) or HSV-1 (H) for 12, 24, and 48 h. Cells were collected and cell lysates were subjected to SDS-PAGE, transferred to membrane, and then immunoblotted with the indicated antibodies.

Because IL-4 can also induce activation of ERK MAPK pathway [21,22], we reasoned that ERK MAPK signaling might be activated in HSV-1-infected BCBL-1 cells. To this end, Western blot analysis was performed to detect activation of ERK MAPK pathway. We found that HSV-1 infection of BCBL-1 cells increased phosphorylated c-Raf, MEK1/2 and ERK1/2 at 12, 24, and 48 h when compared to Mock-infected group (Figure 6).

**Figure 7:**
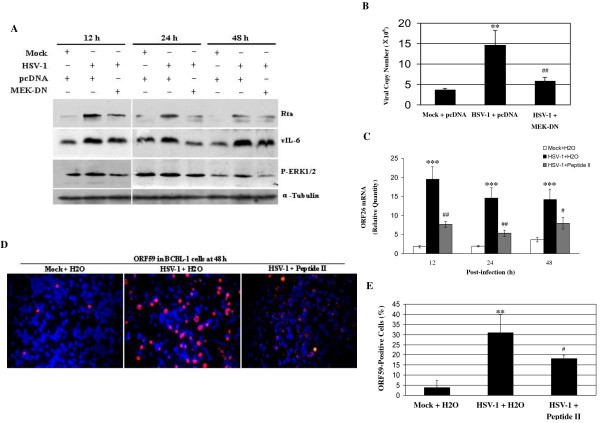
**ERK MAPK pathway partially contributes to HSV-1-induced KSHV replication**. (A) Western blot analysis was used to detect the expression of KSHV Rta, vIL-6 and phosphorylated ERK in MEK-DN or control vector transfected and HSV-1 infected BCBL-1 cells as indicated. (B) Real-time DNA-PCR was used to detect the copy number of KSHV progeny virions in the supernatant of MEK-DN or control vector transfected and HSV-1 infected BCBL-1 cells as indicated. ** *p *< 0.01 and ^## ^*p *< 0.01 for Student's t-test versus Mock + pcDNA and HSV-1 + pcDNA groups, respectively. (C) RT-qPCR was used to detect relative quantities of ORF26 mRNA in peptide II pretreated, HSV-1 infected BCBL-1 cells as indicated. *** *p *< 0.001 for Student's t-test versus Mock + H_2_O group; ^# ^*p *< 0.05 and ^## ^*p *< 0.01 for Student's t-test versus HSV-1 + H_2_O group. (D) KSHV lytic proteins ORF59 expression in peptide II pretreated, HSV-1 48 h infected BCBL-1 cells was detected by IFA staining with ORF59 mAb. Original magnifications, × 10. (E) Quantification of results in D. ** *P *< 0.01 and ^# ^*P *< 0.05 for Student's t-test versus Mock + H_2_O and HSV-1 + H_2_O groups, respectively.

To evaluate the role of ERK MAPK pathway in KSHV replication, MEK-DN, the dominant negative form of MEK1/2, was first used. Western blot analysis demonstrated that control plasmid pcDNA alone did not affect KSHV reactivation by HSV-1, but transfection of MEK-DN lowered HSV-1-induced KSHV Rta and vIL-6 expression through the inhibition of phosphorylation of downstream kinase ERK1/2 (Figure 7A). Next, real-time DNA-PCR was utilized to quantitatively detect the copy number of KSHV progeny virions. It was indicated that the copy number of KSHV virions in the supernatant from MEK-DN-transfected and HSV-1 infected BCBL-1 cells was significantly decreased compared to the corresponding control (Figure 7B). Further, peptide II, an ERK-specific inhibitor, was added to BCBL-1 cells culture before HSV-1 infection. The results from RT-qPCR indicated that ORF26 mRNA in HSV-1-infected BCBL-1 cells pretreated with peptide II was decreased 2.56-fold at 12 h, 2.73-fold at 24 h, and 1.78-fold at 48 h, respectively, when compared to HSV-1-infected BCBL-1 cells pretreated with H_2_O (Figure 7C). Similarly, the results from IFA demonstrated that treatment of peptide II of HSV-1-infected BCBL-1 cells significantly decreased KSHV ORF59 proteins expression (Figure 7D and 7E).

These observations collectively suggest that ERK MAPK pathway also contributes to HSV-1-induced KSHV replication.

## 4. Discussion

Deregulation of cellular signal pathways is involved in the infection process and replication of many viruses and is also likely to contribute to pathogenesis and viral oncogenesis. Many signal pathways, such as JAK/STAT, PI3K/AKT, MAPK, protein kinase C (PKC), nuclear factor kappa B (NF-κB) and Notch have been shown to participate in KSHV infection, replication and angiogenesis [5,23-29]. In this study, we did not observe any evidence that JAK1/STAT3 and JAK1/STAT6, which were the traditional pathways activated by IL-10/IL-10R and IL-4/IL-4R, were involved in KSHV replication by HSV-1, but PI3K/AKT and ERK MAPK pathways induced by IL-10 and IL-4 contributed to this replication.

PI3K/AKT signaling pathway plays an important role in cell growth and survival. PI3K is a heterodimer composed of a catalytic subunit p110 and an adaptor/regulatory subunit p85 [30]. PI3K activation leads to AKT activation. AKT is a critical regulator of PI3K-mediated cell survival and AKT phosphorylates and inactivates several proapoptotic proteins including GSK-3β [31]. PTEN is a negative regulator of PI3K/AKT pathway [32]. PTEN counters the effects of PI3K and inhibits AKT. PTEN is inactivated by phosphorylation, leading to the activation of AKT. With respect to KSHV and activation of PI3K/AKT, many studies focused on viral G protein-coupled receptor (vGPCR) and K1 genes. PI3K/AKT pathway played an essential role in vGPCR sarcomagenesis [33,34]. The activation of PI3K/AKT pathway by K1 promoted cell survival and immortalization and might contribute to KSHV-associated tumorigenesis [35,36]. In this study, we have provided direct experimental evidence that not only suppression of PI3K/AKT signal pathway, but also overexpression of PTEN and activation of GSK-3β inhibited HSV-1-induced KSHV replication, implying complicated functions of PI3K/AKT pathway not only in viral oncogenesis. Interestingly, a report showed that inhibition of PI3K pathway did not impair induction of KSHV lytic replication by metabolic end products of Gram-negative anaerobic bacteria [37]. Another study demonstrated that inhibition of PI3K/AKT pathway enhanced KSHV and murine gammaherpesvirus-68 (MHV-68) lytic replication [38]. We speculated that there were at least three reasons: (1) different inducers and cell lines may exhibit different mechanisms and effects, (2) PI3K and AKT both have a wide range of cellular targets and show complicated functions dependent on the context, and (3) we also simultaneously used dominant negative protein expression plasmids of this pathway, while Peng *et al*. just only used chemical inhibitors. Since chemical inhibitors are known to have pleiotropic effects, the use of dominant negative protein expression plasmids is of value. In addition, authors of these two studies detected only the effects of inhibition of PI3K or AKT on the reactivation of KSHV in PEL cell lines, but the upstream and downstream effectors were not shown.

MAPK cascades are key signaling pathways involved in the regulation of cell proliferation, survival and differentiation. It is not surprising that many viruses including KSHV target MAPK pathways as a means to manipulate cellular function and to control viral infection and replication. Studies from Gao's group demonstrated that ERK, c-Jun N-terminal kinase (JNK) and p38 multiple MAPK pathways had general roles in regulating the life cycle of KSHV by mediating both viral infection and switch from viral latency to lytic replication [39,40]. Among three major MAPK pathways, ERK MAPK pathway has particularly been the subject of intense research in cancer treatment [41]. Because of the fact that KSHV can cause malignancies, KSHV researchers pay more attention to ERK MAPK pathway. There were some reports which focused on activation of ERK MAPK and KSHV replication. For instance, Ford *et al*. demonstrated that inhibiting B-Raf/MEK/ERK signaling by using MEK-specific inhibitors or siRNA construct targeting B-Raf restrained 12-*O*-tetradecanoylphorbol-13-acetate (TPA)-induced KSHV lytic replication [42]. Cohen *et al*. also showed an essential role of ERK signaling in TPA-induced reactivation of KSHV by using MEK-specific inhibitors [43]. Yu *et al*. revealed that Raf/MEK/ERK pathway mediated Ras-induced KSHV reactivation and the same pathway also mediated TPA-induced KSHV reactivation and spontaneous reactivation in PEL cells, by screening expression of a mammalian cDNA library [44]. A more recent study also showed that alloferon inhibited lytic reactivation of KSHV through down-regulation of ERK [45]. Here, we demonstrated a consistent result that activation of ERK signaling partially contributed to HSV-1-induced KSHV replication.

## 5. Conclusions

In summary, we have showed that not JAK1/STAT3 or JAK1/STAT6 but PTEN/PI3K/AKT/GSK-3β and ERK MAPK signal pathways partially contributed to HSV-1-induced KSHV replication. These findings provided further insights into the molecular mechanism controlling KSHV lytic replication and shed light on the pathogenesis of KSHV-induced malignancies.

## Authors' contributions

DQ, NF and WF carried out the experiments. DQ drafted the manuscript. XM, QY and ZL participated in Western blot and IFA. YZ and JZ participated in discussion in preparing the manuscript. CL designed the study and revised the manuscript. All authors read and approved the final manuscript.
